# BUILDING BRIDGES TOWARD WORKFORCE DIVERSITY: STRATEGIES TO SUPPORT RESILIENT LEARNERS

**DOI:** 10.1093/geroni/igae098.0318

**Published:** 2024-12-31

**Authors:** Rebecca Allen, Timothy Ly, Flora Ma, Brian Carpenter, Jennifer Moye

**Affiliations:** University of Alabama, Tuscaloosa, Alabama, United States; University of Alabama, Tuscaloosa, Alabama, United States; Stanford University School of Medicine, Stanford, California, United States; Washington University in St. Louis, St. Louis, Missouri, United States; VA Boston Healthcare System, Boston, Massachusetts, United States

## Abstract

The Building Bridges national virtual conference, held in March 2021, aimed to provide opportunities for psychologists to discuss the dramatic geropsychological workforce shortage, identify means to address this shortage, and discover opportunities and challenges in ongoing efforts to diversify this workforce moving forward. Fifty-six individuals at all stages of training completed a pre-conference survey about workforce diversification and identified advertising and outreach (12.6%), student and faculty recruitment (11.5%), and culturally aware mentorship (10.3%) as strategies for recruiting diverse individuals into geropsychology. Four active workgroups identified enhancing diversity awareness and cultural humility as the most important target areas for change. Qualitative memos from these workgroups were analyzed by a two-member coding team working with an expert in qualitative analyses. Across workgroups, eight emergent themes included: 1) need for financial support, 2) recruitment efforts for diverse students and faculty, 3) intentional awareness emphasizing strength in diversity, 4) creation of inclusive environments, 5) creation of safe spaces for honest and proactive dialog, 6) creation of intersectional and intergenerational learning opportunities, 7) outreach and networking opportunities, and 8) didactic and experiential training. The prioritization of these themes varied by workgroup, with the academia workgroup prioritizing intentional awareness and creation of safe learning environments and the post-licensure clinical workgroup prioritizing recruitment of diverse learners and faculty as well as networking. Strategies for implementing changes to practice that support resilient and diverse learners at all stages of training will be discussed.

